# Kinetics and Thermodynamics of Lactose Mutarotation through Chromatography

**DOI:** 10.1021/acs.iecr.3c04110

**Published:** 2024-03-18

**Authors:** Silvio Trespi, Marco Mazzotti

**Affiliations:** Institute of Energy and Process Engineering, ETH Zurich, 8092 Zurich, Switzerland

## Abstract

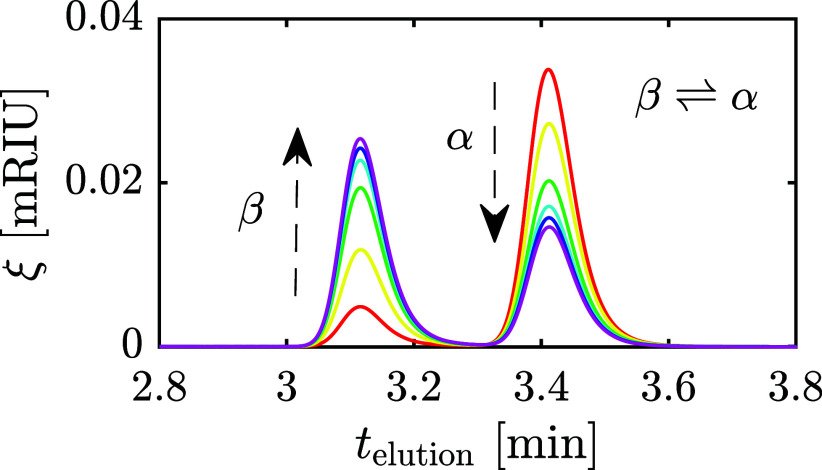

The mutarotation kinetics and thermodynamics of the reaction α-lactose ⇌ β-lactose have been measured in dilute solutions using liquid chromatography without any derivatization step, using a C18 column and pure water as the mobile phase. The effect of temperature (0.5–45 °C) of the starting powder composition (α-lactose-rich or β-lactose-rich powder) and of the solvent composition (water with up to 35% weight fraction of seven organic solvents) has been experimentally investigated. Increasing the temperature leads to faster kinetics, following an Arrhenius model, and to slightly decreasing concentration-based equilibrium ratio. Conversely, increasing the weight fraction of organic solvent at 25 °C resulted in slower kinetics and smaller concentration-based equilibrium ratio. The starting powder composition is shown not to influence the kinetics or thermodynamics of the process. The corresponding parameter estimation problem is thoroughly discussed, taking into account the small difference in response factors of the lactose diastereomers.

## 1 Introduction

Lactose, also known as “milk sugar,” is a disaccharide consisting of a galactose and a glucose moiety. In aqueous solution, lactose undergoes an intramolecular reaction leading to two diastereomers (also known as anomers), α- and β-lactose, which slowly interconvert via a reaction known as mutarotation until equilibrium is reached. The recovery of lactose occurs through crystallization from waste dairy streams and is used in several food and pharmaceutical formulations. The thermodynamically stable solid forms at ambient conditions in aqueous solution is α-lactose monohydrate but, depending on the operating conditions, different crystalline structures can be produced, namely, anhydrous stable α-lactose, anhydrous unstable α-lactose, anhydrous β-lactose as well as cocrystals comprising both α- and β-lactose in different stoichiometric proportions.^1,2^ Each solid form has different physicochemical properties that ultimately impact its performance in their different application area.^3^ For instance, lactose is a very well-known excipient in pharmaceutical applications: due to its greater flowability, α-lactose monohydrate is mostly used as a diluent and carrier in dry powder inhaler (DPI) formulations. On the other hand, anhydrous β-lactose is preferred for direct-compression tabletting.^2^ However, due to the stereoisomerism in the liquid phase, it is challenging to crystallize a powder that is pure in one anomer only; indeed, α-lactose monohydrate contains traces of anhydrous β-lactose whereas anhydrous β-lactose, usually produced by crystallization above 93.5 °C, contains from 15 to 30 wt %/wt of anhydrous α-lactose.^2,4,5^ Despite it being reported^6^ that the anomeric ratio β/α influences the release of active pharmaceutical ingredients deposited on lactose particles, the exact value is rarely measured and a recent study by Altamimi et al.^4^ has shown that commercially available lactose powders differ greatly in terms of anomeric ratio. Schiele et al.^7^ discuss the applicability of ATR-FTIR as an inline technique to monitor the anomeric ratio of lactose in an aqueous solution, whereas Hargreaves^8^ compared different solution-based analytical techniques to determine the anomeric ratio of lactose solutions, namely, gas–liquid chromatography (GLC), polarimetry, ^1^H NMR, and ^13^C NMR:

- GLC has been employed by Dwivedi and Mitchell^9^ to measure the anomeric equilibrium ratio of lactose in an aqueous solution as well as the anomeric ratio of several commercially available powders. A derivatization step in a pyridine/DMSO mixture is required to make lactose sufficiently volatile, while the solvent composition has to be carefully optimized to facilitate the dissolution of the powder and to minimize any change in anomeric composition during the derivatization step due to mutarotation.
- Polarimetry is an effective technique if only two optically active species are present; however, monosaccharide impurities in lactose, like glucose, introduce a systematic error in the measurement. Roetman and Buma^10^ estimated the equilibrium anomeric ratio in an aqueous solution using the specific rotations determined by Buma and van der Veen,^11^ who used GLC to correct the measurement from the traces of impurities.
- NMR spectroscopy is a very powerful analytical technique that has been widely applied to characterize the anomeric ratio of a lactose powder.^4,12^ No derivatization step is required since the anomeric protons in a ^1^H NMR spectrum appear as well-resolved doublets and resonate downfield (4–6 ppm) compared to the ring protons (3–4 ppm). In principle, the anomeric ratio can be measured by integrating the anomeric proton peaks but a series of precautions have to be taken. First, the residual water peak appears between 4 and 5 ppm, exactly the region where the anomeric protons resonate. A common solution is to change the temperature of the measurement to shift the residual solvent peak away, or by suppressing the solvent peak. However, Hargreaves^8^ reported that solvent suppression pulse sequences affect the area of the anomeric protons surrounding the area of interest, thus making the quantification step less straightforward. Alternatively, a freeze-drying step is required to remove the water from the aqueous solution, followed by dissolution in DMSO for an accurate measurement.^13^ In addition, isotope effects cannot be excluded a priori, because of the use of deuterated solvents. ^13^C NMR has been investigated by Hargreaves,^8^ but it is less quantitative than ^1^H NMR due to relaxation and nuclear Overhauser effects and requires a larger number of scans due to the smaller isotopic abundance of ^13^C.

We have recently discussed the applicability of HPLC analysis to reliably estimate the composition of a reversibly reacting mixture,^14^ with particular focus on aqueous lactose solutions. The aim of this article is to apply the chromatographic method with a standard differential refractive index detector to estimate the thermodynamic and kinetic parameters of lactose mutarotation. The results represent the first part of our team’s effort in rigorous model-based design of lactose crystallization. The paper is organized as follows: Section 2 describes the experimental setup and the data analysis strategy; Sections 3 and 4 discuss the modeling and the relative parameter estimation framework; Section 5 reports the final results in terms of impact of temperature, of initial starting powder and of solvent composition.

## 2 Materials and Methods

### 2.1 Chemicals

For all experiments, ultrapure deionized water (Milli-Q AdvantageA10 system, Millipore, Zug, Switzerland) has been used. α-Lactose monohydrate (CAS number 5989-81-1, BioXtra, ≥99% total lactose (GC), ≤4%β-lactose), β-lactose (CAS number 5965-66-2, ≥99% total lactose, ≤30% α-lactose), and all the organic solvents have been purchased from Sigma-Aldrich.

It is highlighted that neither commercial lactose powder is 100% pure in its corresponding diastereomer; hence, we refer to them in the paper as α-lactose-rich and as β-lactose-rich powder, respectively.

### 2.2 Experimental Setup and Procedures

The experiments were carried out in a modular HPLC setup (Agilent Technologies 1200 Series), equipped with a differential refractive index detector (optical cell temperature set to 33 °C). The stationary phase is a ReproSil-Pur 120 C18-AQ (250 × 4.6 mm, Dr Maisch, Germany) column, packed with 5 μm silica particles. The mobile phase consists of pure H_2_O and the flow rate and temperature have been set to 1.05 mL min^–1^ and 10 °C, respectively, to minimize the impact of reaction upon chromatographic separation. The method has been proven to introduce negligible error in the subsequent quantification step.^15^ The analytes are the lactose diastereomers dissolved either in H_2_O or in aqueous–organic mixtures. A thermostated tray is used to hold the sample and to inject 10 μL solution.

Isothermal mutarotation experiments were carried out in a 100-mL automated temperature-controlled glass reactor (EasyMax 102, Mettler Toledo, Greifenbach, Switzerland). Aqueous–organic mixtures are used as the solvent and prepared by weighing the two solvents individually before mixing. Because of the heat released, increasing the temperature to at most 33 °C, 15 min are needed to let the reactor equilibrate at 25 °C before starting the experiment. A precise amount of either α-lactose-rich powder or β-lactose-rich powder is dissolved and the reacting mixture composition is monitored via periodic sampling of 0.5 mL solution, filtration through a 0.22 μm membrane, and chromatographic analysis. The sampling time is measured with respect to the initial time corresponding to when the starting powder enters in contact with the solvent mixture. The time required for full dissolution ranged from 30 s to 2 min, by visual inspection.

**Figure 1:**
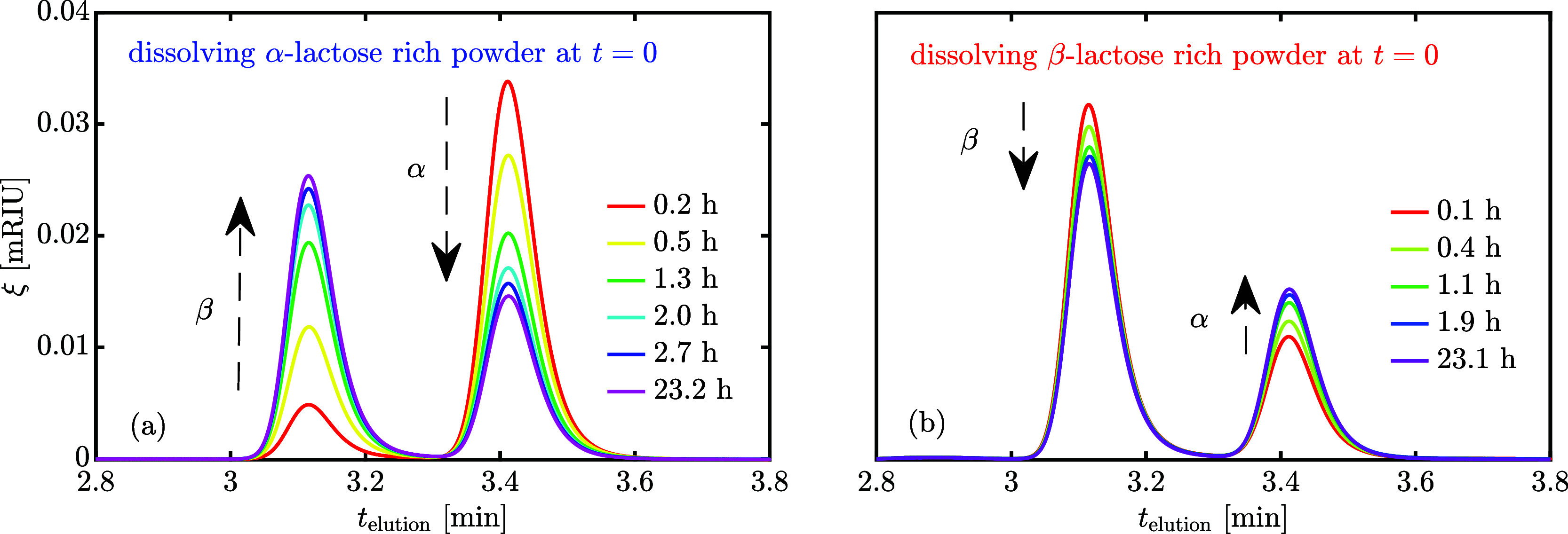
Experimentally measured elution profile of a lactose solution undergoing mutarotation until reaching equilibrium.^14^ Legend reports the time when the elution profile is recorded, measured from the time of full dissolution in water of α-lactose-rich (a) or β-lactose-rich (b) powder. The solution is kept isothermal at 25 °C.

The chromatographic method has an analysis time of 4 min; hence, it is useful for continuous offline monitoring of dynamic processes with sufficiently greater characteristic times, such as the reaction α-lactose ⇌ β-lactose at low to moderate temperatures and the solution crystallization of lactose. To minimize the artifacts coming from sampling and off-line analysis, the HPLC vials used to collect the sample are kept in the fridge at 5 °C. After the sample has been collected, it is placed in the temperature-controlled autosampler at 5 °C and injected within 2 min. The low temperature slows down the isomerization reaction and the chromatography results are deemed representative of the solution composition at the time of the sampling. A complete experimental run consists of multiple elution profiles until the equilibrium of the α-lactose ⇌ β-lactose reaction is reached. Figure 1a,b show the recorded elution profiles when starting from an α-lactose-rich or a β-lactose-rich powder, respectively. Each sample is analyzed with a MATLAB code developed in-house to extract the area ratio of the lactose peaks *A*_R_ = *A*_β_/*A*_α_ and the total area *A*_t_ = *A*_β_ + *A*_α_ as a function of time.

**Figure 2:**
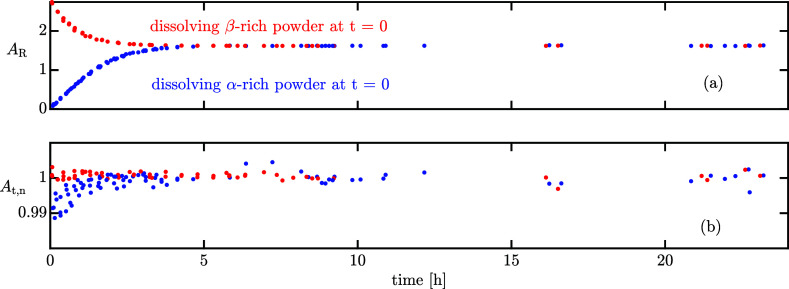
Experimentally measured chromatography ratio of area obtained by peak deconvolution (a) and total normalized area (b) over time for isothermal experiments at 25 °C that start either by dissolving α-rich or β-rich powder.

Figure 2a shows that, independently of the composition of the dissolved powder at *t* = 0, *A*_R_ monotonically approaches the same equilibrium composition from both directions. The mean equilibrium total area, *A*_t,eq_ and the dimensionless total normalized area *A*_t,n_ = *A*_t_/*A*_t,eq_ can be computed (assuming full equilibrium after 8 h); the latter is plotted in Figure 2b. Interestingly, *A*_t,n_ is constant for the β-lactose-rich powder, but increases over time for the α-lactose-rich powder experiments. We conclude that the total area is a function of the liquid phase composition, and the effect is more evident, the larger the variation in composition that the solution experiences. Therefore, although the effect is arguably small, the response factors of the lactose diastereomers are different and cannot be measured accurately as is routinely done by external calibration, since lactose powders are very rarely absolutely pure and undergo mutarotation as they are dissolved in water.

## 3 Modeling

The mass balances for α- and β-lactose include mutarotation rate *r* to describe the dynamic evolution of the composition in a batch system
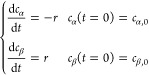
1

Therefore, the total lactose concentration *c*_t_ = *c*_α,0_ + *c*_β,0_ is constant over time. By assuming a first-order reaction rate in both directions, *r* = *k*_α_*c*_α_ – *k*_β_*c*_β_, the analytical solution can be written as
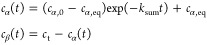
2where *k*_sum_ = *k*_α_ + *k*_β_ and *c*_α,eq_ is the equilibrium concentration of α-lactose. Hence, the anomeric ratio *c*_β_/*c*_α_ can be expressed using eq 2 as
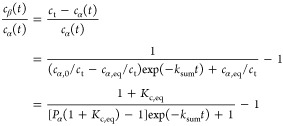
3where the initial powder purity in terms of α-lactose is *P*_α_ = *c*_α,0_/*c*_t_ and the concentration-based equilibrium ratio is defined as *K*_c,eq_ = *c*_β,eq_/*c*_α,eq_ = *k*_α_/*k*_β_. A nonlinear regression can be used to estimate the three parameters involved, namely, *P*_α_, *k*_sum_ and *K*_c,eq_, from a plot of anomeric ratio versus time. The method allows us to estimate both forward and backward rate constants, namely, *k*_α_ and *k*_β_, using the formula
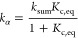
4

## 4 Parameter Estimation Framework

Chromatography does not provide *c*_α_ and *c*_β_ directly, but, rather, *A*_α_ and *A*_β_. Areas and concentrations are linked by the response factor δ, such that, for a linear detector that records a signal at a specified flow rate *F* and injection volume *v*_inj_
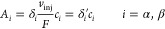
5

Therefore
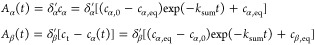
6

Under the assumption of area additivity, *A*_t_ = *A*_α_ + *A*_β_.
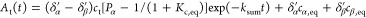
7

The isomerization reaction brings about a change in composition that is reflected in a total area change only if the response factors are different. When the system reaches mutarotation equilibrium, the total area is *A*_t_(*t* → ∞) = *A*_t,eq_ = δ_α_^′^*c*_α,eq_ + δ_β_^′^*c*_β,eq_. By recalling that *c*_t_ = *c*_α_ + *c*_β_ = *c*_α,eq_ + *c*_β,eq_ = *c*_α,eq_(1 + *K*_c,eq_), eq 7 can be rewritten as
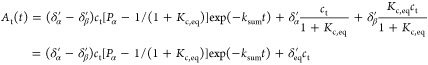
8where δ_eq_^′^ is defined as δ_eq_^′^ = (δ_α_^′^ + *K*_c,eq_δ_β_^′^)/(1 + *K*_c,eq_) and represents the response factor for the solution in mutarotation equilibrium, including the effects of injected volume and flow rate; indeed, *A*_t,eq_ = δ_eq_^′^*c*_t_. Dividing eq 8 by the total area at mutarotation equilibrium, one obtains
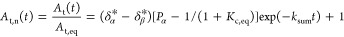
9where δ_α_^*^ = δ_α_^′^/δ_eq_^′^ and δ_β_^*^ = δ_β_^′^/δ_eq_^′^ are the relative response factors, that is, relative to the equilibrium one. It is important to underline that enantiomers would show the same response factors, hence δ_β_^′^ = δ_α_^′^ = δ_eq_^′^ and the total area would show no dynamic evolution over time. For the general case of diastereomers, as for lactose, the relative response factors are different but still related through the concentration-based equilibrium ratio
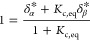
10

Therefore, eq 10 states that δ_β_^*^ can be computed from δ_α_^*^ and *K*_c,eq_. Accordingly, the area ratio is related to the anomeric ratio through the ratio of response factors, that is:
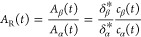
11

Therefore, an analytical expression for the experimentally observable total normalized area and for the area ratio over time is given by eqs 9 and 11, respectively, as a function of four parameters, namely, *K*_c,eq_, *k*_sum_, *P*_α_, and δ_α_^*^. The parameter estimation problem aims to minimize the sum of weighted squared errors, SSE, between the experimentally measured time evolution of the area ratio and of the total normalized area and the corresponding model functions reported in eqs 9 and 11 by changing the value of the parameters θ̲. Since the experimental data are available at discrete times *t*_*i*_, given *i* = 1, ..., *N*_tot_, the optimization problem can be defined as follows
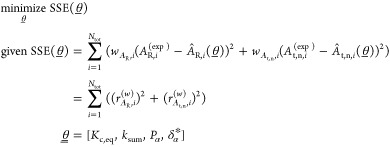
12where the weighted residuals of the area ratio and of the total normalized area,  and , have been introduced using *w*_*A*_R__ and *w*_*A*_t,n__ as weighting vectors.

## 5 Results and Discussion

### 5.1 Mutarotation Kinetics and Thermodynamics at 25 °C Using α-Lactose-Rich Powder

**Figure 3:**
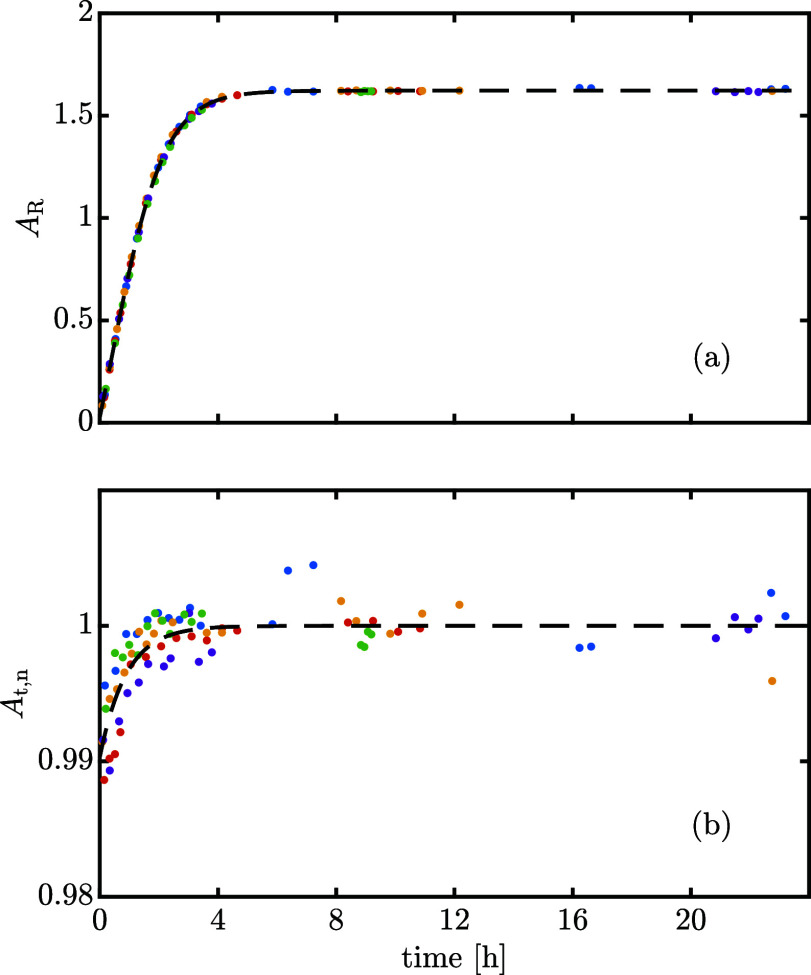
Experimentally measured area ratio (a) and total normalized area (b). The different colors refer to the five different experimental runs. The model optimal response is reported as dashed lines.

Five isothermal mutarotation experiments at 25 °C have been used for parameter estimation, starting from the dissolution of an α-lactose-rich powder: the data set consisting of 84 data points is reported in Figure 3. To accommodate the difference in scale and in precision of *A*_t,n_ over *A*_R_, weighting factors of *w*_*A*_t,n__ ≈ 13 and *w*_*A*_R__ = 1 are assigned. Additional details on the parameter estimation strategy, including the choice of weights and the model adequacy analysis, can be found in the Supporting Information.

The best parameter estimates, the standard errors, SE, and the variance inflation factors, VIF, are reported in Table 1. The standard errors of the parameters were computed using the variance-covariance matrix. In addition, δ_β_^*^ and the forward kinetic constant, *k*_α_, have been computed using eqs 4 and 10 and their standard error is estimated using error propagation analysis. The ratio of the response factors, δ_β_^*^/δ_α_^*^, is also reported.

**Table 1:** Results of Parameter Estimation at 25 °C, Dissolving α-Lactose-rich Powder^a^

parameter	best value	SE	VIF
*K*_c,eq_	1.596	3 × 10^–^^3^	4.8
*k*_sum_ [h^–1^]	1.117	5 × 10^–^^3^	3.7
*P*_α_	0.975	3 × 10^–^^3^	2.9
δ_α_^*^	0.9899	9 × 10^–^^4^	4.7
δ_β_^*^	1.0063	5 × 10^–^^4^	
*k*_α_ [h^–1^]	0.687	3 × 10^–^^3^	
δ_β_^*^/δ_α_^*^	1.017	1 × 10^–^^3^	

a The standard error of the parameters without VIF have been obtained by a posteriori error propagation analysis.

**Figure 4:**
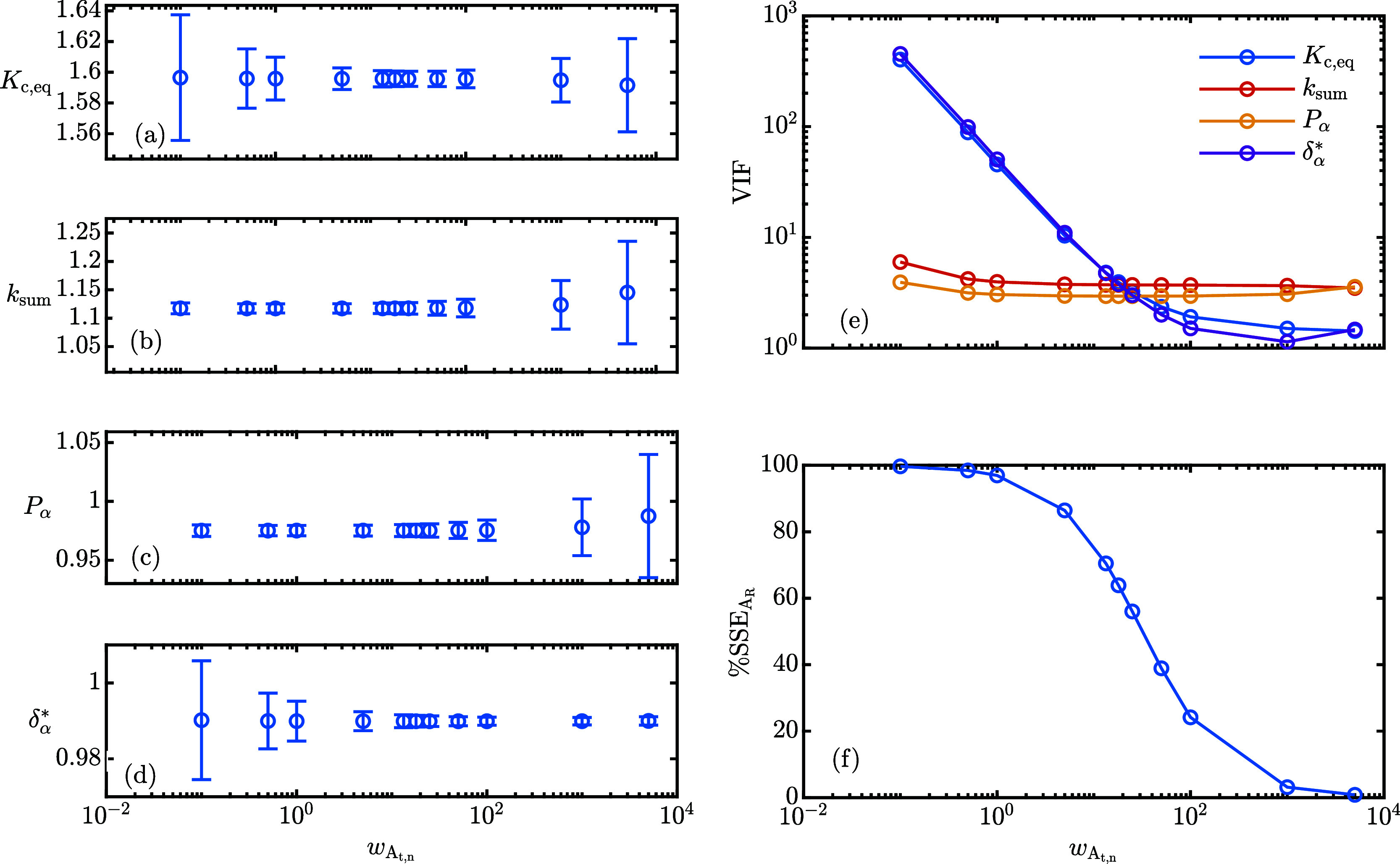
Sensitivity of optimal parameter estimates (a–d) and of the VIF (e) on the weight applied to *A*_t,n_. The error bars refer to the 95% confidence interval. (f) The contribution of  to the objective function.

The VIF are defined as the diagonal coefficients of the inverse parameter correlation matrix and indicate how much the variance of the parameter is increased due to collinearity. Since all of them are very close to 1, no significant parameter multicollinearity is evident from the data analysis, thus leading to small standard errors.^16^ A sensitivity study was conducted to assess the impact of *w*_*A*_t,n__ on the parameter estimation procedure and the results are illustrated in Figure 4.

When *w*_*A*_t,n__ < 1, the fraction of the total sum of squared errors attributable to only , called SSE_A_R__, is close to 100% as shown in Figure 4f. This indicates that the parameter estimation routine is ignoring  and, therefore, significant collinearity between δ_α_^*^ and *K*_c,eq_ arises. This is evident from the very high VIF shown in Figure 4e and from the broad confidence intervals shown in Figure 4a,d. If 10 < *w*_*A*_t,n__ < 100, the collinearity issue is reduced and the confidence intervals are narrow and approximately constant with the applied weight. If *w*_*A*_t,n__ > 1000, only  contributes to the objective function and, therefore, SSE_A_R__ tends to zero. Although δ_α_^*^ is still very well estimated with a narrow confidence interval, since it determines the dynamics of *A*_t,n_, the remaining parameter estimation is troublesome and the confidence intervals broaden considerably. In particular, the purity of the powder *P*_α_ reaches values larger than 1 that are physically impossible. The present study highlights the complex interplay between parameters in a multiresponse system. Equation 11 alone is not enough to accurately determine δ_α_^*^ and *K*_c,eq_ separately since a nonlinear combination of the two parameters characterizes the equilibrium area ratio, hence the collinearity highlighted in Figure 4e. Equation 9 is needed because its dynamics is driven by δ_α_^*^ – δ_β_^*^: combining the two experimental pieces of information allows to significantly reduce the collinearity, but proper weighing is required to find an optimal compromise. Once δ_α_^*^ and, consequently, δ_β_^*^ through eq 10, have been estimated, their ratio can be computed (Table 1) and can be used in eq 11 to estimate *K*_c,eq_, *k*_sum_ and *P*_α_ using ordinary nonlinear least-squares by comparison with the experimental time evolution of *A*_R_. This approach will be exploited in the remaining part of the paper to investigate the kinetics and thermodynamics of lactose mutarotation under different process conditions and starting powder anomeric compositions.

### 5.2 Effect of Starting Powder Composition on Mutarotation Kinetics and Thermodynamics

**Figure 5:**
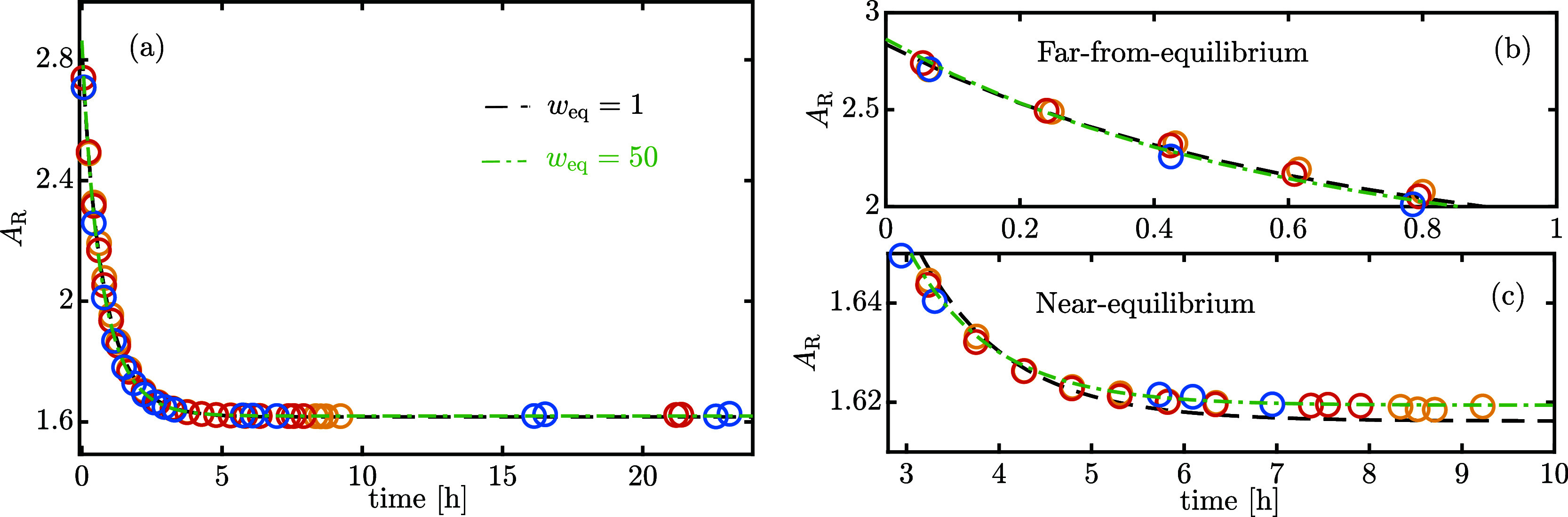
Mutarotation dynamics toward equilibrium dissolving β-lactose-rich powder at 25 °C. Each color refers to one experimental run. The dashed lines correspond to the best fit when no weighting (*w*_eq_ = 1) or optimal weighting (*w*_eq_ = 50) to near-equilibrium points are applied. (b,c) are zoomed-in insets of (a).

The experimental data reported in Figure 2 suggest that the same equilibrium composition is approached either from the β-rich or from the α-rich region, depending on the initial composition of the powder to be dissolved. Since β-lactose-rich powders are commercially available with an unknown amount of α-lactose, the proposed chromatographic method and parameter estimation procedure can be used to accurately measure the anomeric ratio of the powder. By solving the optimization problem reported in eq 12, the fundamental thermodynamic and kinetic parameters, namely, *K*_c,eq_ and *k*_sum_, should be independent of the starting powder anomeric ratio and, thus, be insignificantly different from those already estimated and reported in Table 1. Three isothermal experiments at 25 °C dissolving β-lactose-rich powder have been carried out and the time evolutions of *A*_R_ are shown in Figure 5a. The corresponding values for *A*_t,n_ have already been reported in Figure 2b, but they carry little information because of the smaller change in solution composition compared to that of dissolving α-lactose-rich powder. Therefore, the time evolution of *A*_t,n_ is disregarded for the parameter estimation problem and the ratio of the relative response factors, δ_β_^*^/δ_α_^*^, is assumed known and equal to its best estimate reported in Table 1. Although δ_α_^*^ has been estimated with *w*_*A*_t,n__ = 13, Figure 4 confirms that applying larger weights would change neither the best estimate nor its standard error. Therefore, the present analysis is not influenced by the specific value of *w*_*A*_t,n__ used to estimate δ_α_^*^.

**Figure 6:**
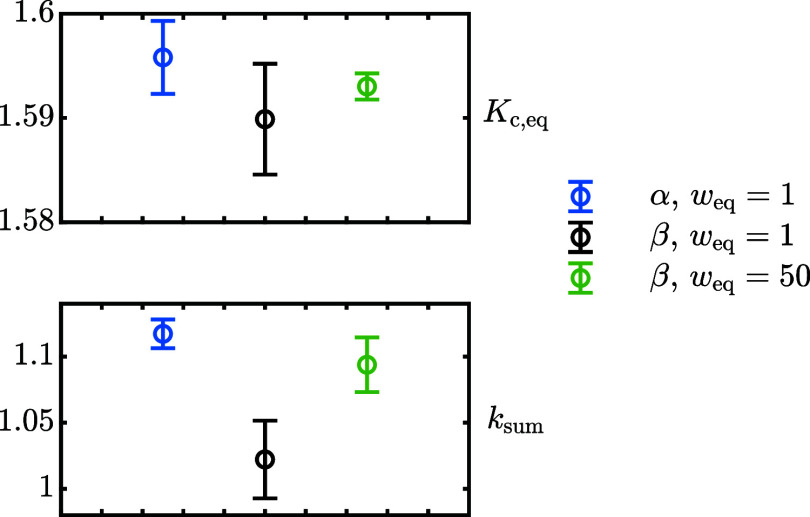
Best estimates for *K*_c,eq_ and *k*_sum_ at 25 °C. The error bars refer to the 95% confidence interval. The legend indicates the experimental data set (α-rich or β-rich, referring to the starting powder anomeric composition) and the weighting factor applied to near-equilibrium-points, *w*_eq_.

**Figure 7:**
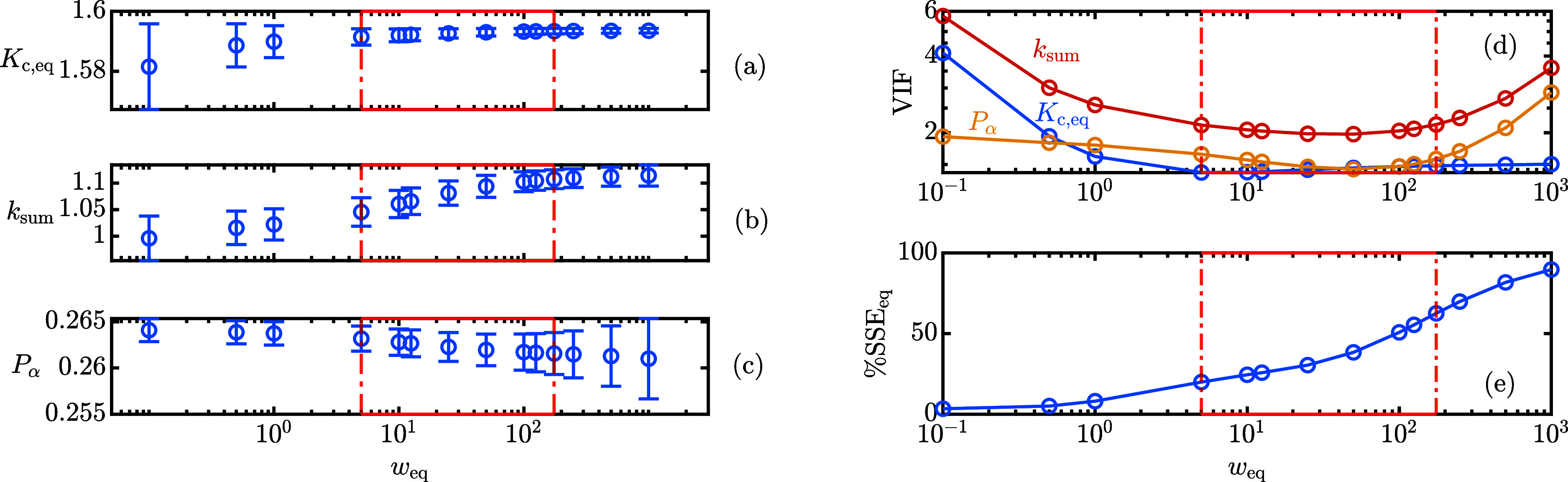
Sensitivity of optimal parameter estimates (a–c) and of the VIF (d) on the weight applied to near-equilibrium points when dissolving β-lactose-rich powder. The error bars refer to the 95% confidence interval. (e) The contribution of *r*_near eq_^(*w*)^ to the objective function. The red rectangle marks the values of *w*_eq_ that lead to normally distributed weighted residuals according to the Jarque–Bera test.

The optimal *K*_c,eq_ and *k*_sum_ according to ordinary least-squares (*w*_eq_ = 1) are reported in Figure 6 as a function of the starting powder anomeric composition, either α- or β-lactose-rich: although *K*_c,eq_ confidence intervals overlap, the β-lactose-rich powder results in a slower mutarotation rate. A closer inspection, however, reveals that the model does not accurately describe the experimental data close to equilibrium (Figure 5c). Since the equilibrium chromatographic area ratio, *A*_R,eq_, seems to be underestimated, a weighting procedure to enhance the contribution of near-equilibrium points to the final objective function is investigated. The data set has been divided, according to the value of *A*_R_, in near-equilibrium points (±5% from *A*_R,eq_, assumed to be reached in 8 h) and in the remaining far-from-equilibrium points. Accordingly, there are two classes of residuals: the weighted residuals of the near equilibrium points are referred to as *r*_near eq_^(*w*)^ and contribute to a fraction of the total sum of weighted squared errors, called the SSE_eq_. A sensitivity study on the impact of the weighting factor applied to near-equilibrium points, *w*_eq_, has been conducted and the results are reported in Figure 7. By increasing *w*_eq_, the near-equilibrium points control the objective function evaluation (Figure 7e) and reduce the confidence interval of *K*_c,eq_, at the expense of *k*_sum_ and *P*_α_ (Figure 7a–c). However, the confidence intervals of the regression parameters are built under the assumption of normally distributed residuals: the red rectangle marks the range of *w*_eq_ that lead to weighted residuals satisfying the Jarque–Bera normality test^17^ (significance 5%). It is interesting to see that the ordinary least-squares solution, namely *w*_eq_ = 1, lies outside this region. By taking *w*_eq_ = 50, around the middle of this region, the confidence intervals for *k*_sum_ overlap (Figure 6). This is the expected result since the composition of the starting powder should not have any effect on the kinetics of mutarotation.

It is not trivial to explain why ordinary least-squares are sufficient when dissolving an α-lactose-rich powder whereas weighted least-squares are needed when dissolving a β-lactose-rich powder. Both processes end at the same anomeric equilibrium composition, corresponding to ca. 1.6 at 25 °C in H_2_O. However, the range of solution composition of the two experiments is very different, as highlighted by Figure 1. Probably, a β-lactose-rich powder that is purer in β-lactose than the one we used in our experiments would not have needed a different weighting procedure for near-equilibrium points. The same sensitivity study has been carried out for α-lactose-rich powder (Figure S5), highlighting that indeed ordinary least-squares satisfy the Jarque–Bera normality test (significance 5%).

Table 2 reports the purity of lactose powder estimated by using the aforementioned chromatographic method. The results are consistent with the manufacturer technical specifications reported in 2.1 that identify only a broad range of composition for the powder. Hence, the proposed method can be applied to accurately estimate the anomeric ratio of an unknown lactose sample.

**Table 2:** Chromatographic Estimation of Purity of Lactose Powders and Comparison with the Manufacturer Specifics, as Reported in Section 2.1

powder	*P*_α_ = *c*_α,0_/*c*_t_	SE_*P*_α__	specifics
α-rich	0.975	3 × 10^–^^3^	>0.96
β-rich	0.262	1 × 10^–^^3^	<0.30

### 5.3 Effect of Temperature on Mutarotation Kinetics and Thermodynamics

**Figure 8:**
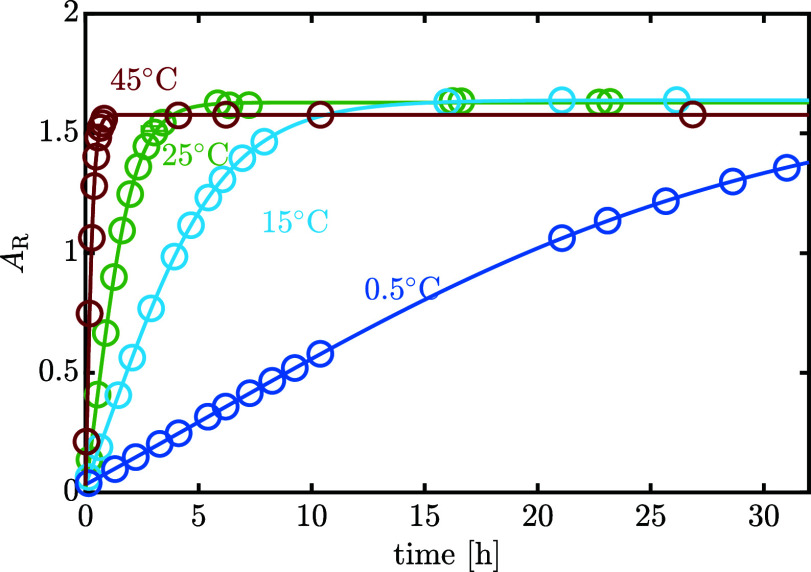
Experimentally measured evolution of area ratio at different temperatures. The solid lines refer to the best model fit.

**Figure 9:**
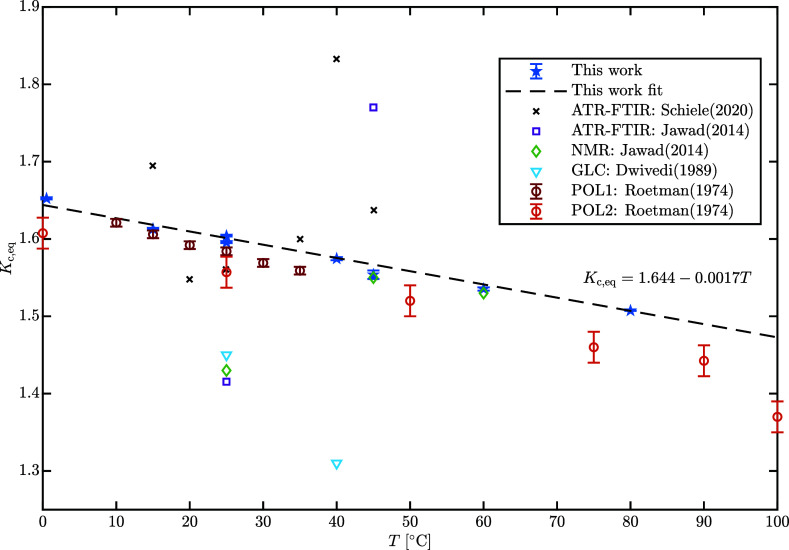
Concentration equilibrium ratio as a function of *T* of lactose in aqueous solution. The error bars refer to ± (SD).

Figure 8 shows how temperature influences the evolution of *A*_R_ when dissolving an α-lactose-rich powder. While the intercept is minimally affected, since it represents the purity of the starting powder, a higher temperature increases the rate at which equilibrium is attained and has a slight impact on the final equilibrium ratio. The estimated concentration-based equilibrium ratio, *K*_c,eq_, is shown in Figure 9 together with data from the literature as comparison. In addition to the kinetic experiments at 0.5, 15, 25, and 45 °C reported in Figure 8, additional isothermal equilibrium measurements of  lactose solutions have been carried out at 40, 60, and 80 °C. Each experiment has been done at least in duplicate.

Figure 9 shows a overview of the available literature data on the concentration-based equilibrium ratio of lactose in aqueous solution, indicating evident differences across the different measurement techniques: this work aligns favorably with the polarimetry-based measurements of Roetman and Buma,^10^ who employed two different methods (POL1 and POL2).

The method “POL1” uses the final equilibrium specific rotation, [θ]_eq_, and the specific rotations of α- and β-lactose, [θ]_α_ and [θ]_β_, measured by Buma and van der Veen^11^ as a function of temperature at 546 nm to estimate the concentration equilibrium ratio as
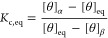
13

The method “POL2” is based on measurements of optical rotation at fixed temperature, and although exhibiting a slightly larger uncertainty, it is consistent with method 1. Overall, chromatography exhibits the same trend as polarimetry but the absolute values are slightly overestimated. Direct analysis with liquid chromatography eliminates the need to carefully purify the lactose diastereomers from optically active monosaccharide impurities like galactose and glucose, since their retention time is between the unretained solute and the lactose peaks, at ca. 2.9 min.

**Figure 10:**
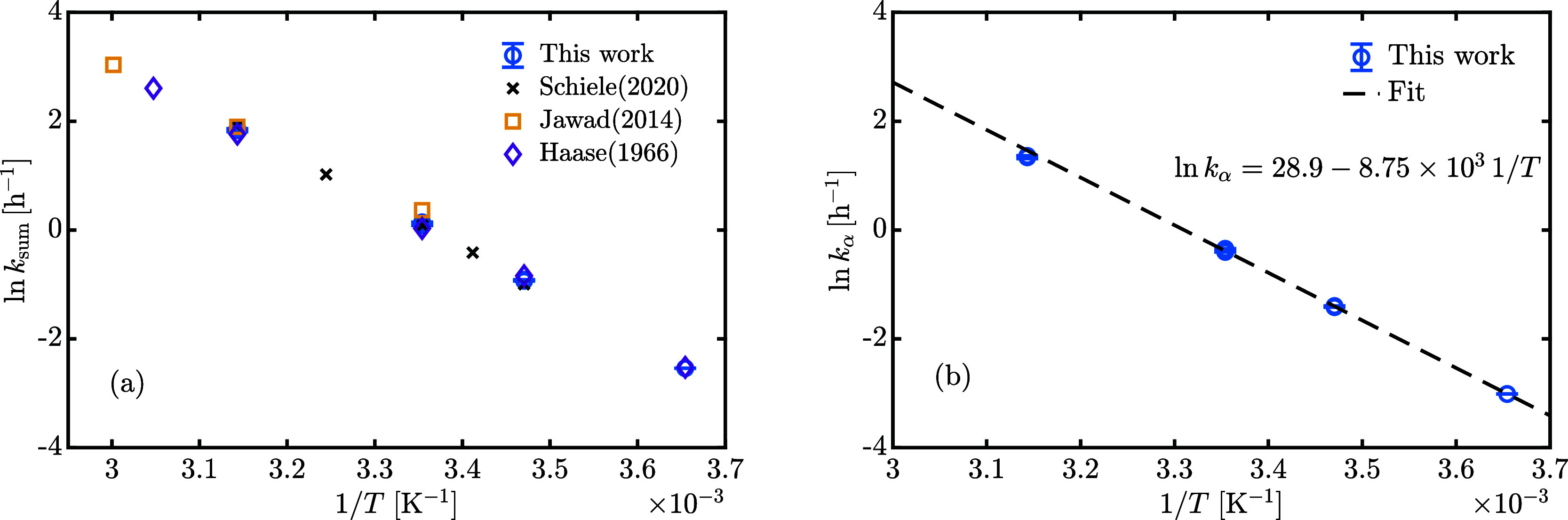
Sum of forward and backward kinetic constants, *k*_sum_, and forward kinetic constant, *k*_α_validity of the Arrhenius exponential

The sum of the forward and backward kinetic constants, *k*_sum_, is shown in Figure 10a. The measured values at 0.5, 15, 25, and 45 °C compare well with literature, and there is significantly less scatter in comparison to equilibrium measurements among different authors, most probably due to systematic errors during calibration that have an influence on the absolute value of β/α at equilibrium and not on its variation over time, as in kinetics. Figure 10b shows the natural logarithm of the forward mutarotation kinetic constant, *k*_α_, as a function of the inverse temperature, estimated using eq 4. The linearity confirms the validity of the Arrhenius exponential model to describe its temperature dependence.

**Figure 11:**
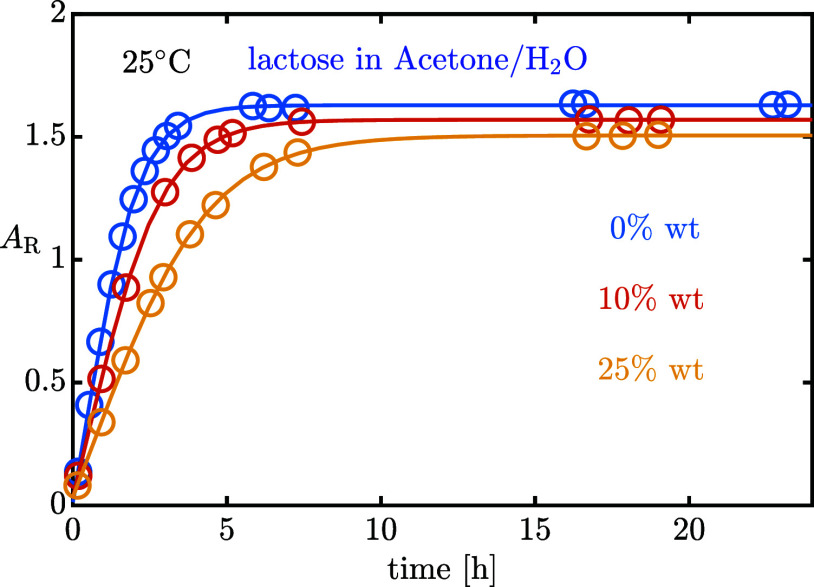
Evolution of *A*_R_ at 25 °C in water/acetone mixture at increasing organic fraction.

### 5.4 Effect of Solvent on Mutarotation Kinetics and Thermodynamics

The same chromatographic analytical method has been used to investigate also the mutarotation rate in aqueous–organic mixtures in order to provide a quantitative assessment of the solvent effect. It is well-known that organics addition slows down the mutarotation rate, but only Majd and Nickerson^18^ investigated quantitatively the phenomenon for lactose in H_2_O–EtOH mixtures. The mutarotation rate of lactose in seven different binary water-organic solvent mixtures [MeOH, EtOH, 1-propanol (*n*PrOH), acetone, DMSO, *N*-methylformammide (NMF), acetonitrile (ACN)] at 25 °C has been investigated in mixtures of 10 and 25% by weight of organic per total weight of solvent. (For EtOH–H_2_O also 35%, corresponding to . For DMSO-H_2_O also 16%.) The presence of a high fraction of organics slightly reduces the retention times of α- and β-lactose during the chromatographic analysis, but the selectivity remains approximately constant. Each elution profile exhibits three distinct peaks: β-lactose, α-lactose, and the organic solvent, in this order. The retention time of each cosolvent is reported in Table 3 and the full elution profiles are reported in Figure S6. The accuracy of the kinetic and thermodynamic measurements is not affected since the organic cosolvent peak is always well separated from the lactose peaks. We tried also formamide as eighth cosolvent, but it eluted between the lactose peaks at 3.25 min, hence saturating the refractive index detector signal and preventing any quantification of the lactose mutarotation within the medium.

**Table 3:** Retention Times (Time of Peak Apex) of the Different Organic Cosolvents at 1.05 mL/min and 10 °C, Using a Pure Aqueous Mobile Phase^a^

co-solvent	*t*_R_ [min]
MeOH	3.6
NMF	4.6
DMSO	4.8
ACN	5.3
EtOH	5.8
acetone	11.5
*n*PrOH	14.6

a β-Lactose and α-lactose elute at 3.1 and 3.4 min, respectively.

By looking at the experimental *A*_R_ evolution for water-acetone mixtures (Figure 11) as a representative example, increasing the organic fraction results in slower mutarotation and in a smaller final concentration equilibrium ratio, namely richer in α-anomer than in the pure water case. The same qualitative conclusions were reported by Majd and Nickerson^18^ for lactose in H_2_O–EtOH mixtures. Several authors have discussed the role of H_2_O solvation of the anomeric carbon in making the β-anomer more stable in solution. This is the case for glucose, maltose and lactose but exceptions to this rule, like mannose, exist and are still not completely understood.^19,20^

**Figure 12:**
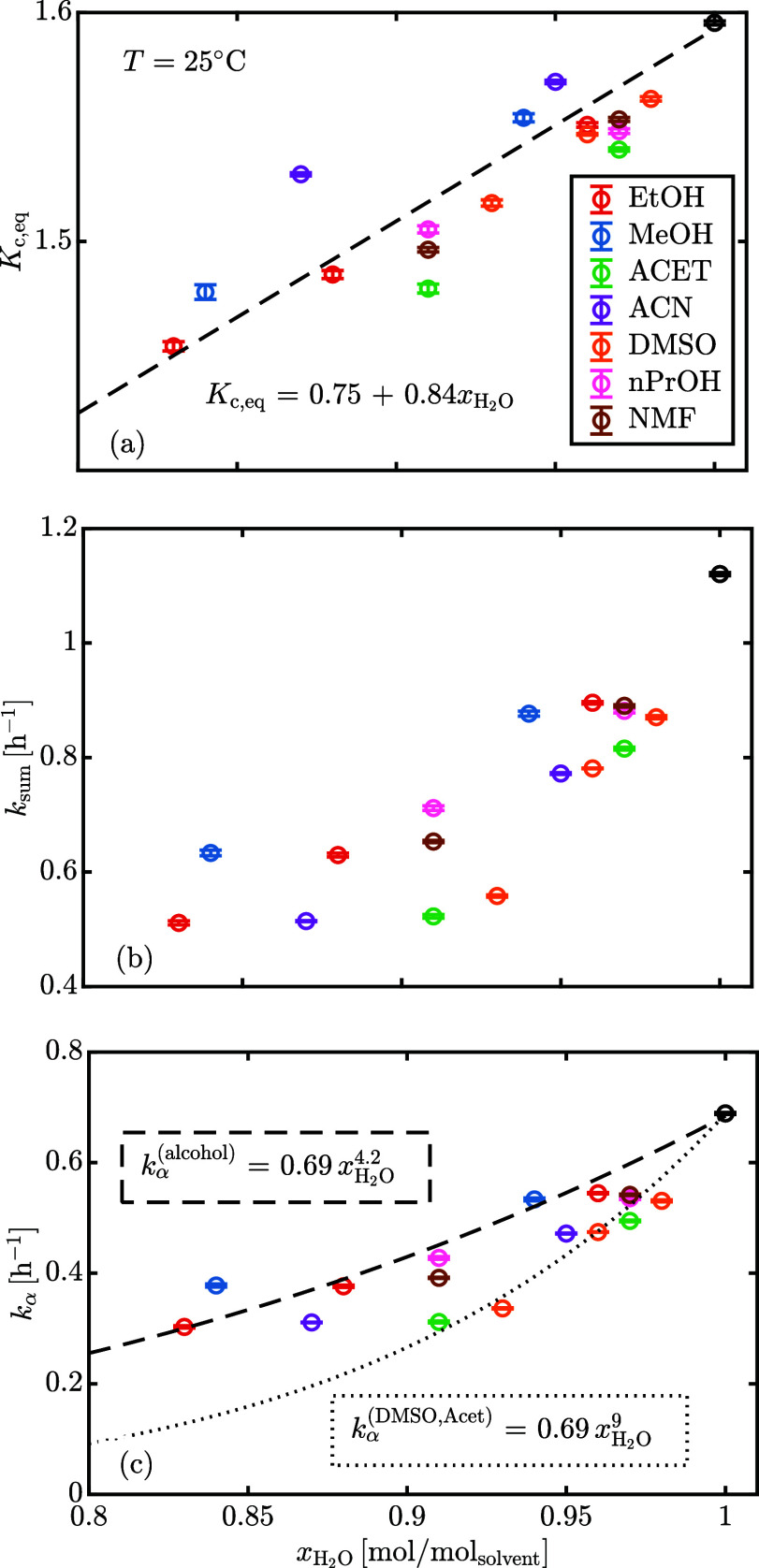
(A) Concentration-based equilibrium ratio, (b) sum of forward and backward kinetic constants, (c) forward kinetic constant as a function of water molar fraction at 25 °C. The error bars refer to ±(SD). The raw data are reported in Table S2.

The equilibrium (*K*_c,eq_) and kinetic (*k*_sum_, *k*_α_) measurements are reported in Figure 12a and Figure 12b,c, respectively, and show a positive correlation with the molar fraction of water in the solvent mixture.

### 5.5 Discussion

Mutarotation is known to proceed through three possible pathways in parallel: acid-catalyzed, water-catalyzed, and base-catalyzed. At pH between 2.5 and 7, the water-catalyzed path is dominating and is responsible for all the reactivity of anomerization.^21,22^Figure 12a shows a linear correlation between the concentration-based equilibrium ratio and the molar fraction of water, supporting the theory of the different solvation tendencies of α- and β-lactose.^20^ The interconversion involves a ring-opening step to give the open chain aldehydic intermediate, which is present in negligible concentration,^23^ followed by ring closure. At intermediate pH, the ring-opening occurs in a concerted fashion, with water molecules participating in a cyclic transition state. Hence, it is reasonable to believe that the concentration of water should enter the reaction rate expression. Kjær et al.^24^ investigated polarimetrically the water-catalyzed mutarotation of glucose in aqueous mixtures of 1–4 dioxane, acetonitrile, and DMSO at 30 °C, finding an order of reaction with respect to the molar concentration of water of 2–4 for all the solvents. Since the hydrogen bond structure of the solvent appears important to stabilize the transition state, it is interesting to observe in Figure 12c that alcohols (strong hydrogen bond donors and acceptors) are the cosolvents with the highest rate of mutarotation for a fixed molar fraction of water whereas acetone and DMSO (aprotic solvents, strong hydrogen bond acceptors only) have the smallest rate of mutarotation. Acetonitrile (aprotic) and NMF (protic) show intermediate reactivity.

Under the assumption that cosolvents are completely inert and that the observed pseudo-first order forward reaction rate contains the contribution from water as a power law , the apparent order of reaction with respect to water, *n*, is estimated to be 4.2 for water–alcohol mixtures and 9 for aprotic solvents (Figure 12c). The order is “apparent” because we are neglecting any direct contribution of the cosolvent in the mechanism but rather assume that it acts as a pure diluent that reduces the water concentration. Actually, preferential solvation effects have an effect on the stability of the cyclic transition state. Therefore, more fundamental studies taking explicitly into account the molecular structure of the solvent are needed to rigorously investigate the impact of mixed solvents on the kinetics and thermodynamics of lactose mutarotation.

**Figure 14:**
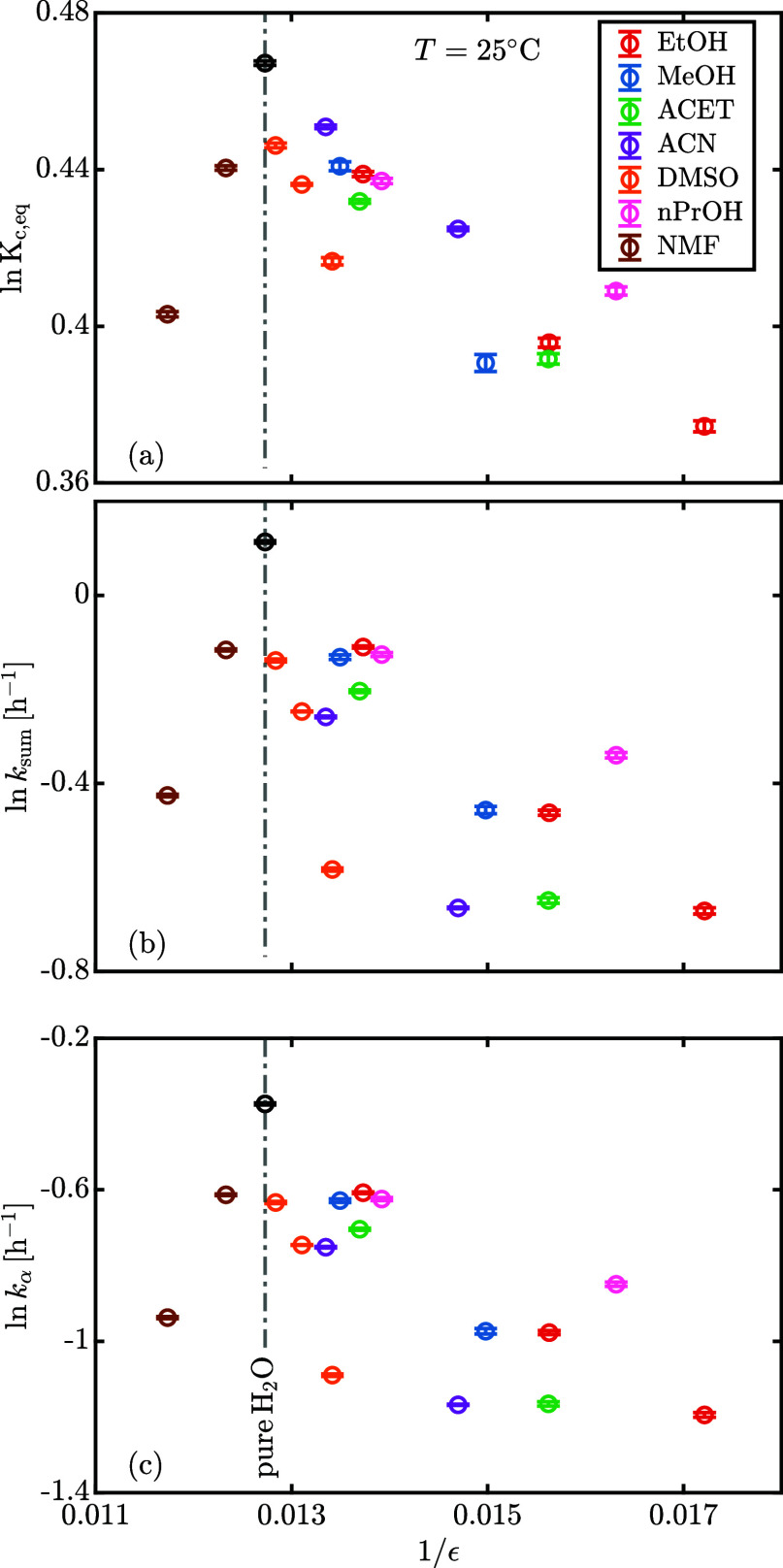
Natural logarithm of (a) the concentration-based equilibrium ratio, (b) the sum of forward and backward kinetic constants, (c) the forward kinetic constant as a function of inverse of the dielectric constant of the solvent mixture at 25 °C. The error bars refer to ±(SD). Dielectric constants for the binary mixtures are from Wohlfarth,^25^ Akerlof.^26^ The raw data are reported in Table S2.

Many theories have been put forward to rationalize the solvent effect on chemical reactions.^27^ One of the first and still widely used is the dielectric continuum model, where the solvent is modeled as a continuous, isotropic, and structureless medium with constant relative static permittivity ε (i.e., dielectric constant). Using Kirkwood’s theory^28^ to take into account the change in mutual electrostatic energy between the solute (either charged or uncharged but with a dipole moment) and the solvent when the solvent composition is varied, a predictive model of kinetic and thermodynamic constants has been derived within framework of the transition state theory,^29,30^ resulting in expressions such as
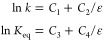
14where the slopes *C*_2_ and *C*_4_ are representative of the differences in charge configuration between reactants and the transition state or the products. A negative *C*_2_ would indicate that the transition state has a higher dipole moment than the reactants, hence the solvents increasing the dielectric constant would stabilize the transition state more and enhance the reaction rate.^29^ We tested the aforementioned theory for lactose mutarotation in mixed solvents: as shown in Figure 14a–c the theoretical linear relationship (eq 14) holds true, but the slope is dependent on the specific solvent mixture. The discrepancies are often ascribed to nonelectrostatic interactions that are not taken into account by the model.^31^ It is common for an aqueous solvent mixture solution to exhibit a decrease in the dielectric constant at increasing organic content. Therefore, we specifically tested NMF-H_2_O mixtures to include in the data set also a mixture whose dielectric constant is higher than pure water, but we discovered that the model is not predictive in this sense: for lactose mutarotation studies, the dielectric continuum model is useful to correlate experimental data but cannot be used to extract any information on the underlying transition state structure from the slope of the experimental curves. It is indeed rare that solvent-sensitive processes show a plain linear correlation with one specific solvent parameter.^27^

## 6 Conclusions

Chromatography has been successfully applied to estimate (i) the kinetic and thermodynamic parameters of a reversibly reacting mixture, namely, a lactose solution undergoing mutarotation, and (ii) the purity of the starting powder. Since the lactose diastereomers exhibit different response factors, the reaction brings about a change not only in the area ratio but also in the total area. The correspondingly needed multiresponse parameter estimation framework has been discussed, and a sensitivity analysis has uncovered how the choice of weights impacts the collinearity between the parameters and, consequently, their confidence interval. The kinetic constant and concentration-based equilibrium ratio for dilute aqueous lactose solutions undergoing mutarotation have been measured as a function of temperature and show a higher degree of precision compared to the literature. The analysis was extended to seven different organic-H_2_O mixtures up to 35% by weight of organic at 25 °C, and the measurements revealed that the reaction rate is the fastest is pure water, thus supporting the hypothesis that water molecules have an important role in transition state structure at intermediate pH. In particular, adding protic solvents like alcohols (MeOH, EtOH, and *n*PrOH) resulted in a higher reaction rate compared to aprotic solvents like DMSO and acetone. Ad hoc experiments with NMF–H_2_O mixtures revealed that the dielectric continuum model could not explain the reactivity trends of different solvent mixtures. These findings are relevant (i) to support or disprove kinetic mechanisms for the mutarotation of sugars in water and mixed solvents and (ii) for the process design of antisolvent crystallization of lactose, since literature data on mutarotation rate of lactose in water-antisolvent systems are scarce.

## Supporting Information Available

Multiresponse weighted least-squares estimation, sensitivity analysis of *w*_eq_ for α-lactose experimental runs, recorded elution profiles of lactose in mixed solvents, summary table of measured thermodynamic and kinetic parameters of lactose mutarotation in mixed solvents at 25 °C (PDF)
