# Supplementary material for: Kinetics and Thermodynamics of Lactose Mutarotation through Chromatography
Source: Ind Eng Chem Res. 2024 Mar 18;63(12):5028–38. doi: 10.1021/acs.iecr.3c04110 (PMC10979398; doi:10.1021/acs.iecr.3c04110)
Supplement: Supplementary file 1 — ie3c04110_si_001.pdf [file ie3c04110_si_001.pdf]

Supporting Information: Kinetics and Thermodynamics of Lactose Mutarotation through Chromatography

Silvio Trespi and Marco Mazzotti*

Institute of Energy and Process Engineering, ETH Zurich, 8092 Zurich, Switzerland

E-mail: marco.mazzotti@ipe.mavt.ethz.ch

1 Additional details on multiresponse weighted least squares estimation

From the experimental data at equilibrium, assumed to be after 8 h at 25 °C in H_2O, it is possible to measure the sample variance $s^2 = \sum(y_i - \overline{y})^2/(N-1)$ of the two measured quantities, i.e. A_R and $A_{t,n}$. The results are reported below in Table S1.

Table S1: measured sample variance at mutarotation equilibrium.

	s^2
A_R	2.92e-5
$A_{t,n}$	2.19e-6

When dealing with a multiresponse weighted least squares problem, Englezos and Kalogerakis [1] suggest to use the information of the relative variance between the quantities to set the weighting matrix: such a choice would lead to maximum likelihood estimates of the parameters. In this case, A_R is around 13 times less precise than $A_{t,n}$, hence in the parameter estimation it has

been decided to set $w_{A_\mathrm{R}} = 1$ and $w_{A_\mathrm{t,n}} = s^2_{A_\mathrm{R}}/s^2_{A_\mathrm{t,n}} = 13$ for each experimental point. This implies that the residuals of $A_\mathrm{t,n}$, $r^{(\mathrm{w})}_{A_\mathrm{t,n}}$, are weighted to play a bigger role in the final objective function evaluation compared to the residuals of A_R, $r^{(\mathrm{w})}_{A_\mathrm{R}}$.

Model adequacy is investigated by looking at the weighted residuals of the model: the normal quantile-quantile plot (Figure S1) exhibits no significant departures from normality and the standardized weighted residuals follow the theoretical straight line of standardized normal residuals, although the tails of the distribution are populated only by $r^{(\mathrm{w})}_{A_\mathrm{R}}$. Indeed, the weighted residuals plot versus time and versus response variable (Figure S2) shows that some $r^{(\mathrm{w})}_{A_\mathrm{R}}$ are slightly above and below the 95% confidence intervals for the residuals when A_R is close to 1. A more detailed analysis (Figure S3) revealed that the excessively high and low residuals belong to the experimental runs with $c_\mathrm{t} = 0.25$ and $1\mathrm{g}/\mathrm{g}_{\mathrm{H_2O}}$, respectively. In these runs, $r^{(\mathrm{w})}_{A_\mathrm{R}}$ exhibit a particularly strong structure superimposed onto the pure random variation. This is not uncommon in dynamic mechanistic models, where the residuals always exhibit a certain degree of correlation.[2] Overall, Figure S2 confirms that the weighted residuals are reasonably homoskedastic and Figure S4 shows that the $r^{(\mathrm{w})}_{A_\mathrm{R}}$ and $r^{(\mathrm{w})}_{A_\mathrm{t,n}}$ values are uniformly scattered around $(0, 0)$, hence supporting the hypothesis that they are uncorrelated.

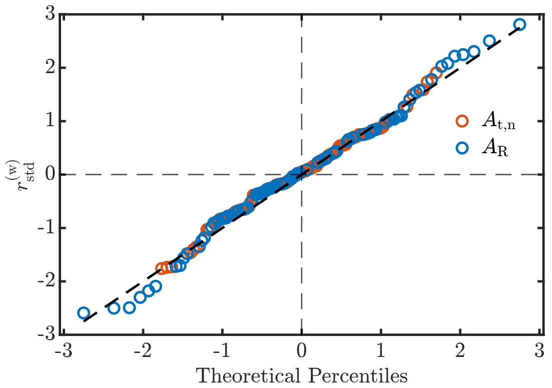

Figure S1: normal quantile-quantile plot of the standardized weighted residuals.

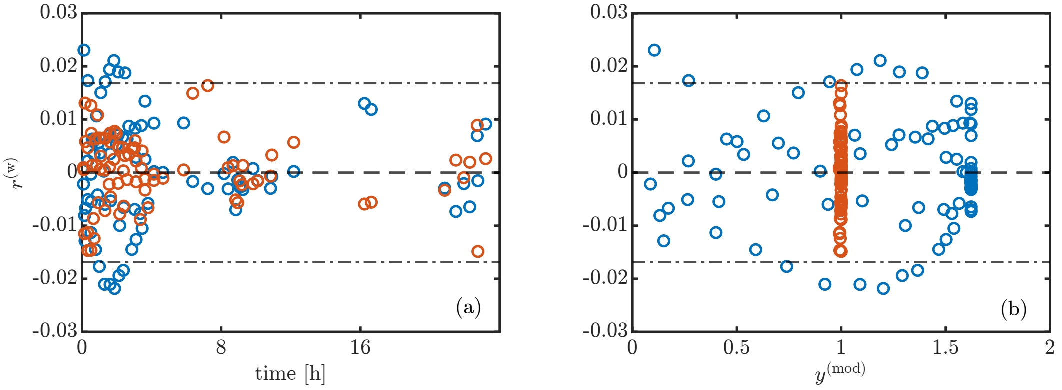

Figure S2: Residuals plot versus time (a) and versus the fitted response value (b). The upper and lower dot-dashed lines correspond to $\pm 1.96\sigma$.

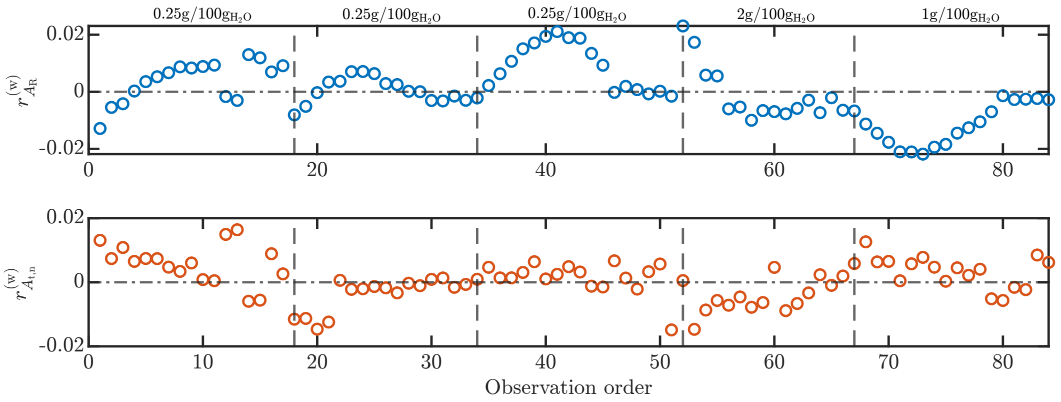

Figure S3: Residuals plot versus observation order for the five experimental runs, separated by horizontal dashed lines. The total lactose concentration in each experiment is reported.

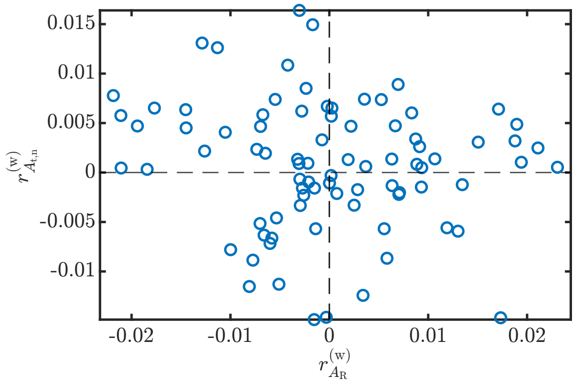

Figure S4: correlation plot between the weighted residuals of the two response variables.

2 sensitivity analysis of w_{eq} for α-lactose experimental runs

Following the same procedure outlined in section 5.2 for β-lactose rich experimental runs, a sensitivity analysis on the w_{eq} has been done also on α-lactose rich experimental runs. The results are reported below in Figure S5. The results confirm that ordinary least squares ($w_{\mathrm{eq}} = 1$) is appropriate as it leads to normally distributed weighted residuals according to the Jarque-Bera test (significance of 5%).[3]

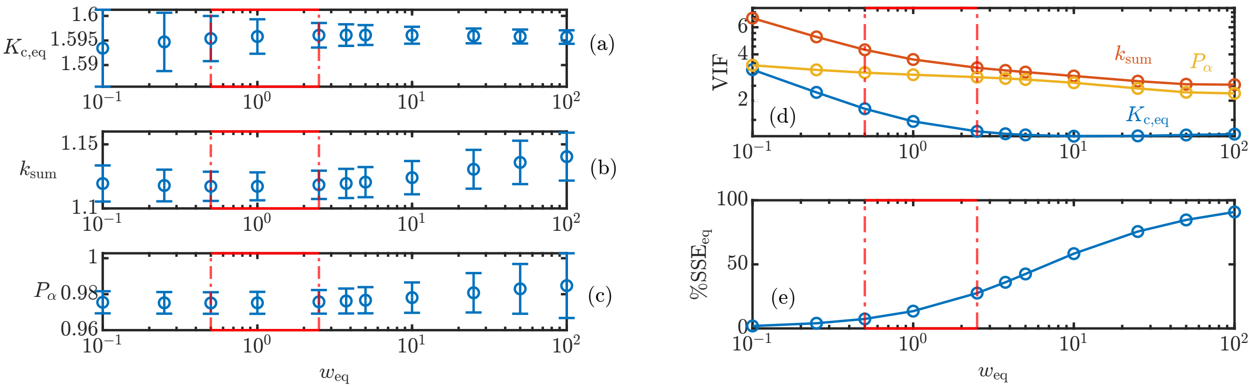

Figure S5: Sensitivity of optimal parameter estimates (a-c) and of the VIF (d) on the weight applied to near-equilibrium points for α-lactose rich experimental run. The error bars refer to the 95% confidence interval. (e) reports the contribution of $r^{(\mathrm{w})}_{\mathrm{near\,eq}}$ to the objective function. The red rectangle marks the values of w_{eq} that lead to normally distributed weighted residuals according to the Jarque-Bera test.

3 Recorded elution profiles of lactose in mixed solvents

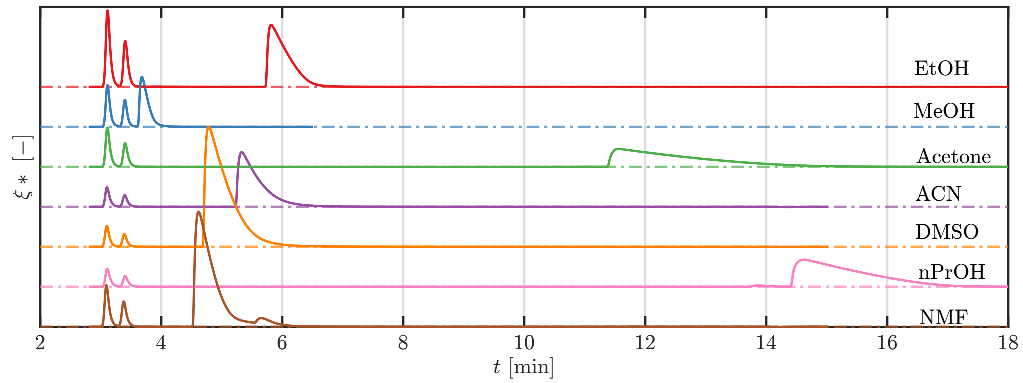

Figure S6: elution profiles of 10/90 wt% organic/H_2O mixtures. The three peaks are, in order, β-lactose, α-lactose and the organic cosolvent. Chromatography operating conditions: 1.05 mL/min, 10 °C. Pure H_2O mobile phase.

4 Summary table of measured thermodynamic and kinetic parameters of lactose mutarotation in mixed solvents at 25 °C

Table S2: weight and molar fraction of organic cosolvent in a water-rich mixture, dielectric constant,[4,5] concentration-based equilibrium ratio and forward kinetic constant of lactose mutarotation. The values reported in the table have been plotted in Figure 12 and 13.

w [$\%\mathrm{g/g_{solvent}}$]	x [$\mathrm{mol/mol_{solvent}}$]	ϵ	$K_{\mathrm{c,eq}}$	k_α [$\mathrm{h^{-1}}$]
10% DMSO	0.025	77.90	1.562	0.531
10% EtOH	0.042	72.83	1.551	0.545
10% MeOH	0.059	74.10	1.554	0.533
10% Acetone	0.033	73.02	1.540	0.494
10% ACN	0.047	74.91	1.570	0.472
10% nPrOH	0.032	71.84	1.548	0.536
10% NMF	0.033	81.10	1.553	0.542
16% DMSO	0.042	76.30	1.547	0.473
25% DMSO	0.071	74.54	1.517	0.336
25% EtOH	0.116	63.98	1.486	0.376
25% MeOH	0.158	66.75	1.478	0.378
25% Acetone	0.094	64.01	1.479	0.312
25% ACN	0.128	68.03	1.529	0.311
25% nPrOH	0.091	61.30	1.505	0.428
25% NMF	0.092	85.22	1.496	0.392
35% EtOH	0.174	58.09	1.454	0.303

The reaction rate is expressed as:

$$r\left[\frac{\mathrm{g}}{\mathrm{g_{solution}\,h}}\right] = k_\alpha c_\alpha - k_\beta c_\beta = k_\alpha c_\alpha\left(1 - \frac{c_\beta/c_\alpha}{K_{\mathrm{c,eq}}}\right) \tag{S1}$$

References

(1) Englezos, P.; Kalogerakis, N. *Applied parameter estimation for chemical engineers*; CRC Press, 2000.

(2) Bard, Y. *Nonlinear parameter estimation*; Academic Press: New York, 1974.

(3) Jarque, C. M.; Bera, A. K. A Test for Normality of Observations and Regression Residuals. *International Statistical Review/Revue Internationale de Statistique* **1987**, *55*, 163–172.

(4) Wohlfarth, C. *Static Dielectric Constants of Pure Liquids and Binary Liquid Mixtures: Supplement to IV/6*; Springer Science & Business Media, 2008; Vol. 17.

(5) Akerlof, G. Dielectric constants of some organic solvent-water mixtures at various temperatures. *Journal of the American Chemical Society* **1932**, *54*, 4125–4139.
